# The autism puzzle: challenging a mechanistic model on conceptual and historical grounds

**DOI:** 10.1186/1747-5341-8-17

**Published:** 2013-11-08

**Authors:** Berend Verhoeff

**Affiliations:** 1 Theory and History of Psychology, University of Groningen, Grote Kruisstraat 2/1, 9712 TS Groningen, The Netherlands

**Keywords:** Philosophy of psychiatry, Autism, Natural kind, Mechanistic property cluster, Demarcation problems, Normativity

## Abstract

Although clinicians and researchers working in the field of autism are generally not concerned with philosophical categories of kinds, a model for understanding the nature of autism is important for guiding research and clinical practice. Contemporary research in the field of autism is guided by the depiction of autism as a scientific object that can be identified with systematic neuroscientific investigation. This image of autism is compatible with a permissive account of natural kinds: the *mechanistic property cluster* (MPC) account of natural kinds, recently proposed as *the* model for understanding psychiatric disorders. Despite the heterogeneity, multicausality and fuzzy boundaries that complicate autism research, a permissive account of natural kinds (MPC kinds) provides prescriptive guidance for the investigation of objective causal mechanisms that should inform nosologists in their attempt to carve autism’s boundaries at its natural joints. However, this essay will argue that a mechanistic model of autism is limited since it disregards the way in which autism relates to ideas about what kind of behavior is abnormal. As historical studies and definitions of autism show, normative issues concerning disability, impairment and societal needs have been and still are inextricably linked to how we recognize and understand autism. The current search for autism’s unity in neurobiological mechanisms ignores the values, social norms and various perspectives on mental pathology that play a significant role in 'the thing called autism’. Autism research needs to engage with these issues in order to achieve more success in the effort to become clinically valuable.

## Introduction

A fundamental question in the philosophy of psychiatry is: What kind of things are psychiatric disorders? This issue is being discussed extensively in a philosophically oriented literature, but there is still no consensus as to the best answer. Can psychiatric disorders best be conceived of as; objects that exist in nature independent of psychiatric classifications (natural kinds, see e.g., [1,2]); scientifically constructed tools or instruments that help to achieve important goals (practical kinds, see e.g., [3]); or maybe as kinds that are brought into being by societies and cultures through the practice of classifying human behavior as distinct kinds (socially constructed kinds, see e.g., [4])?

In this conceptual and historical study, instead of taking an abstract perspective and arguing for a particular model that can be applied to different forms of psychopathology, I focus on autism as a concrete case to explore what kind of thing this specific psychiatric disorder is. Clinicians and researchers working in a particular field are usually not concerned with philosophical categories of kinds. However, I will argue that scientific and clinical thinking about autism implicitly follows a certain model for understanding psychiatric disorders. Autism researchers and clinicians increasingly acknowledge autism’s complex heterogeneity and its dimensionality. Nonetheless, autism research is guided by the depiction of autism as a scientific and physical object that can be discovered and identified with systematic biomedical and neuroscientific investigation. Current assumptions, understandings and practices in the field of autism, I will argue, are compatible with a permissive account of natural kinds, namely the *mechanistic property cluster* (MPC) account of natural kinds recently proposed by Kendler, Zachar and Craver [5] as *the* model for understanding psychiatric disorders in general.

However, despite the attractiveness of a value-free mechanistic model, the MPC model is limited. As recent social and historical studies of autism reveal, there is something profoundly historical and cultural about how autism is diagnosed, delineated and treated. On conceptual and historical grounds, this essay will illustrate that the traditional separation of two types of demarcation problems in (the philosophy of) psychiatry—between distinct mental disorders on the one hand, and between normality and pathology on the other hand—breaks down when considering autism. A mechanistic model of psychiatric disorders only concerns the former demarcation problem while it is indifferent with respect to the latter. Due to this problem, this model is unable to account for the way in which social and cultural norms, and shifting boundaries of normality and pathology shape and transform autism as a psychiatric entity. This study will explore some of the implications of the normativity of autism for defining mental disorder in the DSM-5 [6] and for future autism research.

### Mechanistic property cluster (MPC) kinds: a model for understanding psychiatric disorders

In a recent essay in *Psychological Medicine*, Kendler, Zachar and Craver [5] argue for a mechanistic model for understanding psychiatric disorders. Inspired by the philosopher Richard Boyd [7,8] they suggest that psychiatric disorders can best be viewed as *mechanistic property cluster* (MPC) kinds. Boyd developed the concept of homeostatic property clusters (HPC) to challenge a stringent essentialist model of natural kinds in which a necessary and sufficient property or structure (an essence) directly and causally determines all key features of a kind (Kendler and colleagues replaced the term 'homeostatic’ with 'mechanistic’ to avoid possible confusion due to different meanings of the term 'homeostatic’)^a^.

According to Boyd, there are scientifically important kinds—biological species for instance—that are characterized by a cluster of often co-occurring characteristics and by the underlying mechanisms that bring about their co-occurrence. These clusters do not have invariable and exclusive essences and the members of a kind do not need to overlap in a fixed set of characteristics. Rather, kind membership is defined by some set of empirically discoverable causal mechanisms that explain, in the case of biological species, 'the imperfectly shared and homeostatically related morphological, physiological and behavioral features which characterize its members’ (Boyd [7], p. 142). Similar stable patterns of often complex causal mechanisms that involve interactions between multiple possible levels of explanation—such as physiology, behavior and environment—instantiate the imperfect co-occurring characteristics of the members of a species. They are considered imperfect because 'kind definitions must conform to the (sometimes messy and complex) causal structure of the world’ (Boyd [7], p. 143). Members of a species need not share all their characteristics, and differences between species can be vague. However, this doesn’t imply that there are no stable explanatory mechanisms to be discovered underlying common characteristics of individual members of a species.

Kendler, Zachar and Craver [5] suggest that Boyd’s HPC model of kinds should be the key model for understanding what kind of things psychiatric disorders are. They ask us to consider a multi-dimensional matrix that reflects human mind/brain states. The properties included in this matrix may include genes, neural systems, psychological states, symptoms themselves and environmental inputs. They argue that there are only a finite number of mind/brain states that 'are cohesive and temporally stable, some proportion of which represents 'psychiatric syndromes” ([5], p. 1147). For them, psychiatric disorders are best conceived of as sets of symptoms that are connected through a system of causal mechanisms. Ultimately, these causal mechanisms are what define and sustain the disorder.

This MPC model of mental disorders is attractive for several reasons. It corrects an empirically inadequate 'gene X causes disorder Y’ (essentialist) model, it is compatible with the multicausality, fuzzy boundaries and heterogeneity of most psychiatric disorders, and it provides (unlike pragmatist models) prescriptive guidance for the investigation of objective causal structures that will inform psychiatric nosology in the attempt to carve nature at its joints [5]. These joints are not located at the boundaries of single genes, infective agents or local lesions, but at the boundaries of causal mechanisms [9]. Thus, the MPC model facilitates the prospect of discovery and 'true’ delineation of specific disorders. Since MPC kinds are grounded in the natural features of the world and are 'not merely imposed upon the world by psychiatrists through their classificatory practices’, psychiatric categories will become scientifically valuable in terms of prediction, explanation and control. Even though there is no single causal mechanism or essential property that explains all the superficial properties of a kind, 'the identity of the disease across time and across cultures is grounded in the similarity of the complex mutually reinforcing network of causal mechanisms in each case’ ([5], p. 1147).

Furthermore, as both Kendler, Zachar and Craver [5] and Samuels [10]—who defends an MPC model for delusions—underline, MPC kinds allow 'that the same cluster of symptoms might arise from different etiological, underlying or sustaining mechanisms in different cases’ ([5], p. 1147). There need not be a one-to-one relation between an underlying neurobiological causal mechanism and the resulting cluster of psychiatric symptoms. However, distinct etiological or pathophysiological mechanisms in different members of the same kind must share a similar causal mechanism at another biological level. Biological heterogeneity is allowed, as long as more homogeneous mechanisms can be identified at other biological levels. As we will see, much of the research in autism is, despite profound genetic heterogeneity, directed at identifying unifying neural mechanisms that underlie all—or a subgroup of—autism cases. According to Samuels [10], identifying such unifying mechanisms comprises perhaps *the* fundamental explanatory challenge for an MPC approach to psychiatric disorders. Kendler, Zachar and Craver [5] conclude that we are 'far from being able to define plausible stability-producing mechanisms for most psychiatric disorders’ (p. 1148). However, I will argue that contemporary researchers in the field of autism generally, but usually unknowingly, follow the prescriptive guidance of the MPC model.

### Autism as an MPC kind^b^

Present-day autism researchers and clinicians increasingly emphasize the heterogeneity of autism [11]. Lord and Jones [12] recently stated that 'the most significant scientific challenge to the concept of autism as one “disease” or even “diseases” is the heterogeneity of the genetic findings’ (p. 491). Even though autism is considered to be 'among the most heritable of all mental disorders’ [13] and reviews estimate the heritability of autism to be more than 90% [14,15], the search for autism genes turned out to be extremely complex. Identified single-gene syndromes, such as fragile X syndrome, Rett syndrome and tuberous sclerosis, are assumed to account for approximately 5% of all autism cases [16]. Another 5% of autism cases have been associated with genetic metabolic disorders, such as phenylketonuria and mitochondrial disorders. For the remaining cases, recent whole genome studies [17,18] further affirmed the enormous genetic heterogeneity in autism. These studies illustrate that there are many genetic mutations associated with autism that are very rare. The number of genes associated with autism may be a couple hundred or more, of which the most common mutations were found in just over 1% of the children with autism, and not exclusively in children with autism (see [19,20]).

In addition, Lord [21] pointed out that 'anyone who has met more than one person with an ASD is struck by the differences between these individuals’ (p. 166). However, heterogeneity at both the neurobiological level and the phenotypical level has not resulted in a decomposition of the idea of autism. Despite intense discussions about whether to create a single *autism spectrum disorder* (ASD) category or maintain distinct categories such as Asperger’s disorder, autistic disorder and Rett syndrome in the DSM-5 [22], the idea of a yet-to-be-identified 'true’ autism entity remains pervasive. Wing, Gould and Gillberg [23] state that 'the fundamental problem underlying all autistic conditions and the Triad of Impairments, is absence or impairment of the social instinct present from birth’ (p. 769). Chevallier *et al*. [24] propose a new unifying cognitive theory: 'The social motivation theory of autism’. They argue that autism can be seen as an 'extreme case of early-onset diminished social motivation’ that explains a range of autism characteristics, 'including cascading effects on the development of mature social cognitive skills’ (p. 7).

An essential nature beneath the diversity of apparent signs and symptoms is still assumed to unite the multiplicity of autism. Expressed in terms of a deficit in social intuition or social motivation, and imagined as a fundamental deficit in neurodevelopment, identifying autism is widely presented as a complex scientific challenge, often metaphorically referred to as 'the autism puzzle’ [20]. As the assumed natural entity of autism cannot be identified at a concrete genetic or neuroanatomical level, the attempts to identify autism continue at even more complex and minute molecular and epigenetic levels of neural networks and biological pathways [25].

Geschwind and Levitt [26] suggest a unifying mechanism for autism in which some areas of the brain that normally connect to the frontal lobe are partially disconnected because of disturbances of neural development. Their idea of developmental disconnection, they argue, 'can accommodate the specific neurobehavioral features that are observed in autism, their emergence during development, and the heterogeneity of autism etiology, behaviors and cognition’ (p. 103). Gilman *et al*. [27] and Levy *et al*. [17] suggest that the many different genes associated with autism could play a functional role, via the molecules they express, in a larger biological network that is related to neural development. This larger biological mechanism responsible for neural development (neuronal motility, axon guidance and synaptogenesis) could be disturbed by many *different* molecules expressed by *different* genes that nevertheless lead to *similar* deficits in neural development. These deficits in neural development are in turn thought to underlie the specific symptoms identified as autistic. Sakai *et al.*[28] recently developed a protein interaction network that 'provides a framework for identifying causes of idiopathic autism and for understanding molecular pathways that underpin both syndromic and idiopathic ASDs’ (p. 1). In a similar vein, Voineagu *et al*. [29] provide evidence for convergent molecular abnormalities in ASD, and implicate 'transcriptional and splicing dysregulation as underlying mechanisms of neuronal dysfunction in this disorder’ (p. 380).

A common neurodevelopmental abnormality is still assumed to unite all—or a subgroup of—autism patients. Functional genomics, epigenetics, molecular genetics and systems biology are among the new hopes in the search for autism’s unity. Current developments in autism research fit strikingly well with the MPC model proposed by Kendler *et al.*[5]. Autism researchers and clinicians need to deal with multiple causes and (genetic) heterogeneity, but autism research is nonetheless directed at identifying 'objective’ causal mechanisms that should inform nosologists in their attempt to carve autism’s boundaries at its supposed natural joints. However, a mechanistic approach to autism is limited. This limitation relates to an artificial separation of two familiar types of demarcation problems in psychiatry.

### Two demarcation problems

The first demarcation problem concerns the question of whether and when a certain constellation of signs and symptoms legitimately reflects a distinct category. Is schizophrenia, for instance, a valid disease category and to what extent is schizophrenia distinct from schizo-affective disorder, delusional disorder or any other ('normal’) state or trait? A central term in this debate is *validity*. This is a complex construct with several meanings and subtypes, which I will not discuss here in detail. Rather, I will briefly focus on how this term has been used in psychiatric nosology.

Robins and Guze [30] were the first to propose a formal method to improve the validity of psychiatric categories. In their influential paper on establishing diagnostic validity for schizophrenia, they proposed five phases in the evaluation of a putative diagnostic category that they thought were an indication of its validity: clinical description, laboratory studies, delimitation from other disorders, follow-up studies, and family studies. These validators were used to show that 'apparent “schizophrenia” with a good prognosis is not a mild form of schizophrenia, but is a different illness’ (p. 987). Their findings provided the basis for the distinction between schizophrenia and schizophreniform disorder in DSM-III [31]. Kendler [32] expanded the set of validators and distinguished between antecedent validators (familial aggregation, premorbid personality, and precipitating factors), concurrent validators (including psychological tests), and predictive validators (diagnostic consistency over time, rates of relapse and recovery, and response to treatment).

A common assumption underlying discussions about validity and proposals to increase the validity of psychiatric categories is that a 'truly’ valid psychiatric disorder reflects genuine underlying (pathophysiological) differences in relation to other disorders and normal brain functioning. Kendell and Jablenski [33] argue that while the diagnosis of psychiatric disorders is still based on clinical observation, a distinct syndrome will be valid if we reasonably expect that it can be defined by physiological, anatomical, chromosomal, histological or molecular abnormalities. Besides increasing reliability, since DSM-III the aim of psychiatric classification systems has been to create psychiatric categories that facilitate the identification of genes, neurotransmitter mechanisms and other neurobiological markers related to psychiatric disorders. In line with this aim, the ultimate goal of psychiatric taxonomy, as the research agenda for DSM-5 [34] concluded on this issue, has become 'to translate basic and clinical neuroscience research relating brain structure, brain function, and behavior into a classification of psychiatric disorders based on etiology and pathophysiology’ (p. 70).

The MPC model of psychiatric kinds is in line with this effort. By informing nosologists, the MPC model attempts to increase the validity of psychiatric categories, where validity depends on whether a certain psychiatric category captures genuine underlying differences. The MPC model is supposed to bring us closer to the ultimate goal of current psychiatric nosology, which is a system based on etiology and pathophysiology with neuroscience providing the foundation for classification and possibly individual diagnosis. However, the MPC model and conventional discussions on validity are largely indifferent towards another central demarcation problem in psychiatry. This second demarcation problem which will be discussed below, concerns a more general question: How can the distinction between normal and pathological mental functioning be made?

In a comprehensive monograph on this contested topic, Bolton [35] discusses several possible ways to make this distinction. One way, for instance, is to conceive pathological mental functioning as 'a matter of breakdown of meaningful connections in mental life’ (p. 16). Examples of a breaking down of meaningful connections include emotions that are excessive or have no appropriate object, behavior that is not under the control of the person’s will, and beliefs that have no basis in experience. Another possibility, inspired by the work of Jerome Wakefield [36], is to conceive of pathological mental functioning as 'not functioning as it has been naturally designed to do in the evolutionary process’ ([35], p. 17). Despite the value of some of the theories he discusses, Bolton concludes that there is not one single theory that adequately distinguishes all forms of mental pathology from normality. Furthermore, in line with a widespread consensus among philosophers of psychiatry, Bolton concludes that how the line between what is normal and what is pathological in mental functioning is drawn depends on social, cultural and individual values and circumstances. Even Jerome Wakefield [36], who is considered to be on the naturalist side concerning mental disorders, acknowledges that a biological dysfunction needs to be *harmful* in order to become pathological, and harm cannot be understood independent of sociocultural circumstances.

In defending the value-free MPC approach for delusions, Samuels [10] is well aware of the two potentially conflicting demarcation problems. However, he argues that the normativity of pathology is not necessarily but only contingently connected with delusions. Without some reason to suppose that this connection is a necessary one, this normativity does not pose a threat to the MPC model regarding delusions [10]. Kendler, Zachar and Craver [5] are equally aware of the evaluative nature of mental pathology as they acknowledge that 'values are intimately involved in determining which psychiatric kinds deserve clinical attention’ (p. 1147). However, values are not only involved in determining whether the condition we have come to call autism deserves clinical attention, they are also involved in defining and delineating this psychiatric kind in the first place. Taking the distinction between normality and pathology into account is crucial for understanding the way in which autism emerged, transformed and is currently defined as a diagnostic entity.

### A short history of the concept of autism^c^

Anyone working in the field of autism will agree that the way in which autism is understood today, differs remarkably from how it was initially described by Leo Kanner in 1943 [37]. For Kanner, autism was a rare and severe disorder of affective contact, characterized by two cardinal features: a 'profound withdrawal from contact with people’ and an 'obsessive desire for the preservation of sameness’ [38,39]. Kanner based his findings and definition of 'early infantile autism’ on parents’ descriptions and lengthy clinical observations of a small and selected group of children. Despite immediate professional debates about the possible causes of this new diagnostic entity, Kanner’s first description of autism remained largely unchallenged for approximately the first two decades after its introduction.

From the 1960s on, the concept of autism changed significantly. Influenced by the first epidemiological and experimental studies with autistic children, an important shift in understanding the core features of autism took place. Longitudinal studies [40], for instance, showed that Kanner’s profound withdrawal and disturbance in affective contact tend to lessen considerably as the autistic child grows older, while other symptoms like intellectual disabilities and language problems tend to persist. The first psychological experiments with autistic children (e.g., by Hermelin and O’Connor [41]) that tested intellectual and expressive abilities suggested that language and speech problems were not due to profound affective withdrawal or motivational failure, but instead due to a poor understanding of the meaning of spoken words. Whereas Eisenberg and Kanner ([38], p. 557) regarded 'the vicissitudes of language development’ as derivates of the fundamental disturbance in affective contact, many autism researchers in the 1960s and 1970s argued that the basic defect in autism was the inability to develop a normal use and understanding of language, in combination with a global defect in the integration of other sensory stimuli. Deficits in language, speech and cognition became cardinal features and key characteristics in diagnosing and recognizing autism, and (neuro)psychological tests and experiments became an integral part of the diagnostic process.

Influenced by autism researcher Lorna Wing, a second shift in the conception of autism’s core deficits occurred in the 1980s. An influential epidemiological study [42,43] and the introduction of the work of the Austrian pediatrician Hans Asperger into the Anglo Saxon autism literature [44] contributed to a new perspective on autism. On the basis of this epidemiological study, which investigated the prevalence and co-occurrence of social, language, and cognitive impairments in children, Wing argued for a central division between 'socially impaired’ and 'sociable’ children. This division provided a new basis for categorization, and social impairment–instead of language or sensory deficits–became *the* central distinguishing aspect in the study of autism. In addition, social impairment was no longer understood as Kanner’s 'extreme autistic aloneness’ but as a continuum of problems in social interaction ranging from subtle deficits in the use and understanding of the 'unwritten rules of social behavior’ to profound social withdrawal ([43], p. 42). Furthermore, Wing [44] argued that Asperger’s cases [45] and Kanner’s cases [37] were essentially similar. Despite the variations in terms of severity of impairments, Wing argued that both disorders shared a common and essential characteristic: the impairment of two-way social interaction that 'arises from a lack of ability to understand and use the rules governing social behaviour’ ([44], p. 116).

Today, in line with this second shift, autism is predominantly conceptualized as a spectrum disorder characterized by a range of social and behavioral difficulties that are due to an 'impairment of the social instinct present from birth’ ([23], p. 769). Lord and Jones [12] even conclude that Kanner’s essential 'rituals and insistence on sameness are somewhat less prevalent in ASD, occurring in approximately 25% of a sample of children with ASD’ (p. 496). These changing understandings of the core characteristics of autism are usually understood as better reflections of what autism 'really’ is. Changes in views on autism are generally ascribed to scientific advancements, better epidemiological research and improved psychometric techniques [46]. However, recent historical and sociological approaches towards autism express a different perspective on the changing meaning of autism.

Eyal *et al.*[47] and Nadesan [48] in particular demonstrate that autism and its conceptual changes cannot be understood independently from educational and cultural institutions; ideas about deficiency, abnormal behavior and unmet needs of children; and the need of clinicians, parents, researchers and society at large to demarcate and structure a particular problem with social behavior. Eyal *et al.*[47], for instance, explain the broadening of the criteria for autism and the exponential rise in numbers of people diagnosed with autism in terms of a process of deinstitutionalization of mental retardation in the mid-1970s, a growing availability of services from 1991 onwards when autism was added to the Individuals with Disabilities in Education Act (IDEA) in the United States federal law, and parental activism. The way in which Kanner’s cardinal symptom of autistic aloneness and lack of interest in other people was changed in DSM-III-R [49] into only one of many manifestations of 'qualitative impairment in reciprocal social interaction’, indicates not a better understanding of autism, they argue, but an increased need to demarcate and treat a wider range of problems. Emerging autism therapies, the rise of an active parents’ movement, and new ideas about childhood pathology and 'deviant’ behavior played an important role in this process [19].

In her history of the emergence of autism, Nadesan [48] demonstrates how the formalization of compulsory education and the emergence of the child guidance movement in the first half of the twentieth century led to increased forms of surveillance over childhood, an increased public concern over 'deviant’ children posing a threat to social stability, and, hence, an increased demand for childhood mental health professionals. Understandings of normality and pathology in mental health shifted and community clinics and special schools for deviant children emerged. Autism could only emerge as a diagnostic category because, according to Nadesan (p. 53), 'it was within these schools and clinics that a new cadre of experts … encountered a class of children who escaped the increasingly narrow parameters of normality but whose apparent pathologies could not be satisfactorily explained by the extant psychiatric categories’.

### Diagnosing autism and intercultural variability

As this brief history of the concept of autism illustrates, the core characteristics of autism have changed remarkably. Currently, it is not disturbances of affective contact or language deficits, but impairments in social interaction that are central in defining and diagnosing autism. Throughout these changes, conceptualizing autism has always been related to the medical commitment to correcting undesirable conditions, and defining autism has been related to the needs of clinicians, researchers, society and parents to structure, comprehend and treat different types of disability, suffering and 'abnormality’ wherever they occur [19]. The official and generally accepted *DSM* category of autistic disorder–the starting point for clinical practice and fundamental autism research–is replete with value terms that express these needs and undesirable conditions. For instance, the quintessential *impairments* in social interaction are characterized by a *failure* to develop *appropriate* relationships, a *lack* of seeking to share enjoyment, and a *lack* of social reciprocity. Communication *impairments* are manifested by a *lack* of *appropriate* make-believe or social imitative play, et cetera [50].

A diagnosis of autism inevitably (co-)depends on personal and cultural ideas of appropriate peer relationships, normal play behavior, and appropriate empathizing skills. Diagnostic and classification practices cannot avoid depending on often implicit socio-cultural norms related to normal child development, such as making friends, seeking to share enjoyment or making eye contact, and on experiences of abnormality, impairment and disability that emerge within a social sphere.

When social context and socio-cultural norms do play a role in defining and delineating autism, one would expect that different cultural perceptions of social competence, child development and health will lead to intercultural variability in conceptualizing and diagnosing autism. Existing cross-cultural and multicultural epidemiological studies on autism are not particularly sensitive to this conceptual variability [51] and typically use Western *DSM* definitions and diagnostic tests to investigate differences in prevalence rates across nations, cultures and races (see e.g. [52,53]). Prevalence differences related to racial or ethnic background [54], socioeconomic status [55], neighborhoods [56] or Western and non-Western nations [57] are usually ascribed to differences in awareness, (access to) services, diagnostic resources, available treatments or research methodology. Unfortunately, the common assumption that autism is a recognizable neurobiological disorder with little variation in behavioral manifestation across culture, ethnicity, and social class has made investigating intercultural variation in conceptualizing and diagnosing autism, and in perceptions of abnormality, disability and suffering appear less relevant for cross-cultural autism research.

A study that investigated the prevalence of autism in South Korean school-aged children estimated a prevalence of 2.64% in a population sample [58]. About two-thirds of the children identified in the study as having autism attended mainstream schools and were undiagnosed and untreated. The authors conclude that their findings underscore the need for better autism detection, assessment, and services in South Korea. However, their findings raise an unattended question of whether their Western autism diagnosis is meaningful in a South Korean cultural context where many children apparently function reasonably well with a supposed autism disorder. Major cultural differences between South Korea and Anglo-American society, for instance regarding how children play and socialize, how parents think about normal child play and development and how structure and routine are emphasized within the classroom [59] are not considered to affect autism prevalence rates nor are they considered to be relevant for conceptualizing and diagnosing autism. Cross-cultural prevalence studies typically do not question the Western concept of autism but project it onto different socio-cultural contexts.

A few anthropological studies take a different 'insider’ view of autism from the perspective of local professionals and societal members. Connors and Donnellan [60] for instance explored the perception of disease and disability from the cultural perspective of the Navajos. Their anthropological fieldwork revealed that different expectations, notions of childhood, ideas of social competence, health and sickness, resulted in different ideas of abnormality and autism. Local health professionals tended not to use biomedical disease entities or syndromes like autism, but instead to describe and classify isolated symptoms in relation with the broader context of the harmony of the community. Daley and Sigman [61] analyzed diagnostic conceptualizations of autism among Indian psychiatrists and hypothesized that, compared to the United States, Indian parents and professionals were more likely to detect and emphasize social deficits as fundamental to autism due to the importance of social conformity in Indian culture. Whereas in Anglo-American culture, which is more focused on language development, parents and professionals were more likely to detect and underscore delays or regression in language skills as central in diagnosing autism. Unfortunately, these types of comparative studies are rare, but they illustrate a way in which cultural and professional norms, habits and practices shape diagnostic practices and conceptions of autism.

### Why autism is not an MPC kind

As Cooper [62] convincingly argues, culture-bound syndromes that emerge in highly specific social and historical contexts can still be distinct 'natural’ disorders. For instance, similar to different kinds of igneous rocks that are created under specific environmental conditions, a mental disorder can be influenced by cultural and environmental factors such as diet, lifestyle or environmental pollution, and still be a distinct natural (MPC) kind grounded in a network of causal mechanisms. Social and cultural factors can be considered as causal agents that become part of the entire network of causal mechanisms associated with the particular kind. Biology and culture may interact, Cooper argues, 'so as to produce cases of a disorder that are recognizably and reliably similar to each other and such disorders can usefully be recognized by psychiatric classification systems’ ([62], p. 331).

Following Cooper’s argument, putative culturally and historically specific *causal* factors (e.g., child-rearing practices or environmental toxins) and, as a hypothetical consequence, varying prevalences or manifestations of autism all over the world would not necessarily threaten a mechanistic (MPC) model of autism. However, the fundamental requirement of the model, that the *identity* and *boundaries* of a particular disorder are *set* by causal mechanisms, is particularly problematic for autism. As Kendler, Zachar and Craver argued, 'the identity of the disease … is grounded in the similarity of the complex mutually reinforcing network of causal mechanisms in each case’ ([5], p. 1147). 'An MPC kind’ is their best answer to the ontological question: What kind of thing is a psychiatric disorder? However, the historically and culturally variable boundaries of 'impairment of social interaction’ or 'a lack of ability to understand and use the rules governing social behaviour’—now considered essential features of autism—are clearly not set by causal mechanisms. This issue of setting the boundaries of autism is not just a matter of demarcating a coherent cluster of signs and symptoms, it is also a matter of demarcating normality from pathology.

Social and cultural values and norms not only influence whether a certain cluster of symptoms is considered as a disorder, but they play, in autism at least, a necessary role in what becomes a recognizable cluster of symptoms in the first place. Defining autism as a nosological entity incorporates the (shifting) needs and discontents of a society regarding how an individual interacts with others, empathizes, makes friends, seeks to share enjoyment, initiates small-talk, and figures out implicit social norms. This blurs the boundaries between the two discussed demarcation problems as demarcating autism (and identifying neurobiological dysfunctions related to autism) *necessarily* involves demarcating undesirable conditions. An MPC model of autism that attempts to ground the identity and boundaries of autism in causal mechanisms disregards these normative dimensions.

### Implications for the definition of mental disorder in DSM-5

Both in the definition of mental disorder in DSM-IV [63] and in the proposal by Stein *et al*. [6] for a modified definition of mental disorder for DSM-5, the two discussed demarcation problems are reflected in separate criteria (see also Broome & Bortolotti [64] and Verhoeff & Glas [65]). In particular, criterion A, that a mental disorder is 'a behavioral or psychological syndrome or pattern that occurs in an individual’, implicitly concerns the first demarcation problem, whether a certain cluster of features legitimately reflects a distinct disease ([6], p. 1761). Criterion B—'the consequences of which are clinically significant distress (e.g., a painful symptom) or disability (i.e. impairment in one or more important areas of functioning)’—refers to the second general problem of demarcating normality from pathological mental functioning.

The separation of the two demarcation problems in different criteria is compatible with an MPC model of psychiatric kinds, in which a behavioral or psychological syndrome or pattern (cluster) reflects underlying (psychobiological) mechanisms. Whether it 'deserves clinical attention’ (criterion B) can be approached as a separate issue. However, for autism, as we have seen, these two problems are inextricably linked to each other. This essay is not the place to propose an alternative definition of mental disorder. However, the phrase 'in an individual’ in criterion A is particularly problematic for autism and deserves some more attention here.

As Broome and Bortolotti [64] indicated, the phrase 'in an individual’ is complex, controversial and carries conceptual baggage. It may seem evident that certain psychological states and behavioral patterns belong to or reside in an individual. However, as the case of autism illustrates, the recognition and description of an autistic behavioral pattern or particular autism signs and symptoms is profoundly embedded in a social and cultural context. Defining autism depends on historically and culturally variable ideas about deficiency, abnormality and dysfunction, and on the need to demarcate and treat particular discontents and impairments that have appeared. The case of autism, generally considered to be one of the most 'biological’ of all mental disorders, illustrates Broome and Bortolotti’s [64] suggestion: 'that at the very least the claim that a disorder occurs “in an individual” warrants further examination’ (p. 1784).

For a definition of mental disorder, the challenge will be to find a way to integrate values and social norms that unmistakably play a role in what we have come to understand as mental disorder, without falling victim to a medicalization of social problems (see also Sadler [66]). This may be close to impossible, but it is better to expose and explore this difficulty than to artificially restrict mental disorder to that which is 'in an individual’ while ignoring societal aspects.

## Conclusions

This essay challenges—not with empirical evidence or research, but on conceptual and historical grounds—the mechanistic model that is implicit in current autism research and practice. The mechanistic property cluster (MPC) model [5], which attempts to define and delineate autism in terms of causal mechanisms, is attractive for several reasons: it corrects an empirically flawed essentialist model; it is compatible with the multicausality, heterogeneity and fuzzy boundaries of many mental disorders; it provides prescriptive guidance for the investigation of objective causal structures; and it 'satisfies the intuitions of reductionist psychiatrists’ (p. 1148). Current autism research fits the MPC model strikingly well, as autism research—despite the acknowledged heterogeneity of the condition—is guided and regulated by the depiction of autism as a scientific and natural object that can be discovered and identified with systematic neuroscientific investigation.

However, the MPC model of natural kinds disregards the way in which autism relates to ideas about what kind of behavior is inappropriate and in need of correction or support. Two familiar demarcation problems in psychiatry–between distinct psychiatric disorders on the one hand, and between normality and pathology on the other hand–are unjustifiably disconnected in an MPC approach to autism. As historical studies, a few anthropological studies and current conceptions of autism show, normative issues concerning disability, impairment, normal social interaction and societal needs and discontents have been and still are inextricably linked to how we recognize and understand autism.

### Some implications for autism research

The current tenacious search for autism’s unity in objective neurobiological mechanisms pays little attention to the values, social norms and context-dependent perspectives on mental pathology that play a significant role in 'the thing called autism’. For instance, issues like the social acceptance of diversity; the framing effects and performativity of an autism diagnosis regarding a child’s identity, social relations and societal challenges and possibilities; the relationship between suffering and impairment and the demands of the social world; the historical development of the concept of autism; and implicit (shifting) norms in social life and children’s behavior, all become relatively unimportant in the scientific search for autism’s unity at neurobiological levels. A serious engagement of clinicians and autism researchers with these issues will enable the autism field to broaden its scope in the direction of the human and social sciences. After all, as Patil and Giordano ([67], p. 6) point out, 'the distinction between what is normal and abnormal … will need to be made, and any such distinction must be practical in the sense of its viability to sustain the good of patient-centered clinical care’. Therefore, these contextual and social issues need to be taken into account in order to better inform clinical practice, and whether neurobiological autism research 'will serve such practical ends remains to be seen’ ([67], p. 7).

This does not imply that neuroscientific research could not be valuable in autism research. The identification of neurobiological mechanisms associated with clusters of symptoms will remain important in developing potential treatments. However, letting go of an MPC model for autism and the idea of discovering the true nature of autism in causal mechanisms de-inevitabilizes current biomedical perspectives and research purposes, and creates space for alternative modes of classification that for instance do not take autism as a nosological (neurobiological) entity as the main point of departure for research and clinical practice. Furthermore, de-emphasizing the objective to ground autism in causal mechanisms might result in a broadening of the scope of interventions, allowing them to go beyond the (neurobiological) individual. Eventually, what we should aim for is not grounding autism in causal mechanisms, but creating a sensible connection between autism research and the everyday concerns and needs of 'autistic’ patients and their families.

## Endnotes

^a^The term 'natural kind’ is not at all clear-cut and different accounts of natural kindhood have been associated with many different classes of things, such as chemical elements, biological species, colors, stars, and psychiatric disorders. Some philosophers question the value of the term [68] and it is not my intention to get drawn into metaphysical disputes about what exactly defines a natural kind. However, a natural kind approach matters in scientific practice. Natural kinds make good objects of scientific discovery, are related with the search for mechanistic explanations, and allow for inductive generalizations [19].

^b^This sections draws on [19] pages 416-421.

^c^For a longer history of the concept of autism, see [69].

## Competing interests

The author declares that he has no competing interests.

## Author’s information

Berend Verhoeff is a Psychiatrist and Philosopher of Science. Currently he is working as a PhD candidate at the Theory and History of Psychology Department at the University of Groningen in the Netherlands. His doctoral research is a historical and philosophical analysis of the genesis and development of autism as a nosological entity.

## Authors’ contributions

BV is responsible for the full content of the manuscript.
